# Protein-Protein Interactions in Crystals of the Human Receptor-Type Protein Tyrosine Phosphatase ICA512 Ectodomain

**DOI:** 10.1371/journal.pone.0024191

**Published:** 2011-09-15

**Authors:** María E. Primo, Jean Jakoncic, Martín E. Noguera, Valeria A. Risso, Laura Sosa, Mauricio P. Sica, Michele Solimena, Edgardo Poskus, Mario R. Ermácora

**Affiliations:** 1 Consejo Nacional de Investigaciones Científicas y Técnicas (Conicet), Ciudad Autónoma de Buenos Aires, Argentina; 2 Cátedra de Inmunología de la Facultad de Farmacia y Bioquímica, Idehu, and División Endocrinología del Hospital de Clínicas J. de San Martín, Universidad de Buenos Aires—Conicet, Ciudad Autónoma de Buenos Aires, Argentina; 3 Photon Science Directorate, Brookhaven National Laboratory, Upton, New York, United States of America; 4 Departamento de Ciencia y Tecnología, Universidad Nacional de Quilmes, Bernal, Buenos Aires, Argentina; 5 Paul Langerhans Institute Dresden, Molecular Diabetology, Universitätsklinikum “Carl Gustav Carus”, University of Technology Dresden, Dresden, Germany; 6 Max Planck Institute of Molecular Cell Biology and Genetics Dresden, Dresden, Germany; Consejo Superior de Investigaciones Cientificas, Spain

## Abstract

ICA512 (or IA-2) is a transmembrane protein-tyrosine phosphatase located in secretory granules of neuroendocrine cells. Initially, it was identified as one of the main antigens of autoimmune diabetes. Later, it was found that during insulin secretion, the cytoplasmic domain of ICA512 is cleaved and relocated to the nucleus, where it stimulates the transcription of the insulin gene. The role of the other parts of the receptor in insulin secretion is yet to be unveiled. The structures of the intracellular pseudocatalytic and mature extracellular domains are known, but the transmembrane domain and several intracellular and extracellular parts of the receptor are poorly characterized. Moreover the overall structure of the receptor remains to be established. We started to address this issue studying by X-ray crystallography the structure of the mature ectodomain of ICA512 (ME ICA512) and variants thereof. The variants and crystallization conditions were chosen with the purpose of exploring putative association interfaces, metal binding sites and all other structural details that might help, in subsequent works, to build a model of the entire receptor. Several structural features were clarified and three main different association modes of ME ICA512 were identified. The results provide essential pieces of information for the design of new experiments aimed to assess the structure *in vivo*.

## Introduction

Protein-tyrosine phosphatases (PTP) and the counteracting kinases have a central role in the regulation of cell division, growth, differentiation, and metabolism [1]. PTP can be either cytoplasmic or transmembrane receptor-like proteins (RPTP) and participate in cell-cell and cell-matrix contacts, possess a diversity of adhesive and multimerization modules [2], and are involved in human diseases such as cancer, autoimmunity, and degenerative processes [1].

ICA512, (also known as PTPRN, IA-2 or PTP35), and phogrin (also known as PTPRN2, IA-2 β or IAR), are homologous RPTP chiefly expressed in secretory granules (SG) of brain, pituitary, pancreatic islet, and adrenal endocrine cells and were originally identified as major autoantigens in type-1 diabetes mellitus [3], [4]. Although the molecular details are poorly understood, these proteins are involved in hormone and neuropeptide secretion: knockout mices lacking one or both proteins exhibit glucose intolerance, lowered insulin secretion, and abnormal secretion of pituitary hormones and female infertility [5], [6].

During SG maturation in β-cells, lumen proteins experience significant changes in their environment: H^+^, Ca^2+^ and Zn^2+^ concentrations increase progressively as insulin hexamers aggregate and crystallize [7]. Concomitantly processing by furin-like hormone convertases produces mature ICA512 [8], which comprises extracellular (residues 449–575), transmembrane (residues 576–600), and cytoplasmic domains (residues 601–979).

The ICA512 cytoplasmic domain tethers SG to actin microfilaments through the formation of a complex with β2-syntrophin and utrophin [8]. Glucose stimulation of insulinoma cells prompts the CDK5 mediated phosphorylation of β2-syntrophin, thereby weakening the association of ICA512 with β2-syntrophin and thus facilitating the mobilization and exocytosis of SG [9]. The ensuing transient insertion of ICA512 in the plasma membrane, in turn, triggers the calcium–dependent, calpain-1 mediated cleavage of the cytoplasmic domain, which is then targeted to the nucleus, where it promotes transcription of insulin and other SG genes [8].

Most RPTP have two cytoplasmic catalytic domains, one of which is inactive. It has been proposed for RPTPα that oxidation of one of the cysteine residues in the catalytically active domain by reactive oxygen species (ROS) induces a conformational change coupled to the formation of disulfide-linked dimers [10], [11]. Interestingly, the conformational change is coupled to conformational changes in the ectodomain [12]. Also, the transmembrane regions possess dimerization potential per se [13], and, conceivably, as part of the signaling processes, the whole receptor may undergo coupled conformational changes during association or binding to yet unknown ligands. Although ICA512 and phogrin have only one and inactive cytoplasmic domain they homo- and heterodimerize [8], [14]. In this regard, formation of heterodimers could account for the reduced enzymatic activity of RPTPα upon co-expression with ICA512 or phogrin [14].

Recently, we reported the X-ray structure of residues 468 to 558 of the mature extracellular domain of ICA512 at pH 8.5 and 1.30 Å resolution [15]. We found that this fragment has a ferredoxin-like fold and is related to the SEA (Sea-urchin sperm protein, Enterokinase, Agrin) family of proteins [16], which are specialized domains for oligomerization and interaction with the extracellular matrix.

The finding that ICA512 possesses an extracellular adhesion module unified and simplified the classification of the RPTP family. In fact, with the single exception of the subtype R7 (PCPT1), all RPTP are now characterized by the inclusion of a large variety of extracellular modules apt for the interaction with the extracellular matrix and cell-cell contact [1], [2], [17].

Residues 468 to 558 of ICA512 comprise most of the mature ectodomain (residues 449 to 575). Unfortunately, residues 449 to 467 and 559 to 575 are excised upon incubation at pH 7–8 by autoproteolysis [15]. Also, attempts to crystallize the full mature ectodomain at acidic pH, a condition that prevent autoproteolysis, have failed. These circumstances impede the X-ray characterization of the crucial segment 559–575, which connects ME ICA512 to the transmembrane region and defines the topological orientation of this domain at the cell surface.

Since an experimental solution to the above problems seems at the moment to be beyond reach, theoretical modeling is a suitable alternative to move forward in the characterization of the receptor. To this end, however, more information about the ectodomain region that can be crystallized is needed. Particularly, it would be desirable to know if this region undergoes pH-induced conformational changes, binds metals through sites harbored in its structure, and dimerizes.

To address the above issues, we prepared new crystals of ME ICA512 in various conditions. We also crystallized a variant designed to weaken one of the association modes proposed previously and which does not dimerize in solution ([15], Primo and Ermácora, unpublished work). The X-ray structures of the crystallized proteins were solved and several important structural details were clarified.

## Materials and Methods

### Miscellaneous

Unless otherwise indicated, reagents were from Sigma (St. Louis, Missouri). Changes in solvent accessible surface area (ASA) upon dimerization were calculated as Δ*ASA*_AB_ = *ASA*_A_+*ASA*_B_−*ASA*_AB_. Size exclusion chromatography was carried out at 20°C using an AKTA FPLC system and a Superose 12 column (GE Healthcare, Uppsala, Sweden) equilibrated with 20 mM sodium phosphate, 150 mM NaCl, pH 7.0.

### Protein expression and purification

The fragment of ICA512 crystallized for this work comprises residues 470 to 558 (UNP Q16849, PTPRN_HUMAN). The preparation of corresponding DNA for expression in *E. coli* was carried out by PCR with appropriate primers, and protein purification was as described before [18]. Identity and integrity of the protein product were verified by mass analysis, which yielded the value expected from the sequence within 1 Da.

### Crystallization

Crystals of meIA-2, were obtained after 2 weeks at 19°C with the hanging-drop method. The reservoir solution (300 µl) was 30% (w/v) PEG 4000, 0.2 M CaCl_2_, and either 0.1 M Tris-HCl, 0.1 M HEPES, or 0.1 M acetic acid/sodium acetate, at pH 8.5, 7.5 or 4.5, respectively. The drop (4 µl) was a 1∶1 blend of reservoir and protein solution (∼10 mg/ml in 50 mM NaCl, 10 mM Tris-HCl, pH 7.4).

### Data collection and processing

X-ray diffraction data were collected at the National Synchrotron Light Source (NSLS) on beam line X6A, at 100 K, using an ADSC Q270 detector (Area Detector Systems Corp., Poway, CA). Before data collection, crystals were soaked in mother liquor supplemented with 10% (w/v) PEG 400 and flash-cooled in liquid nitrogen. Relevant data-collection parameters are given in Table 1.

**Table 1:** Data collection, phasing and refinement statistics.

**Sample information**				
ME ICA512 variant	S508A	S508A	wild type	wild type
pH	7.5	8.5	8.5	4.5
PDB entry	3N4W	3NG8	3N01	3NP5
**Data collection** a				
Synchrotron	NSLS	NSLS	NSLS	NSLS
Wavelength (Å)	1.0332	1.0332	0.9537	0.9537
Resolution (Å)	20.00–1.45	20.00–1.35	20.00–1.30	20.00–1.80
	(1.47–1.45)	(1.37–1.35)	(1.32–1.30)	(1.83–1.80)
Space group	P2_1_2_1_2_1_	P2_1_2_1_2_1_	P2_1_2_1_2_1_	P4_1_
Unit cell parameters (Å)	a = 31.47	a = 31.55	a = 31.49	a = b = 44.66
	b = 66.02	b = 66.54	b = 66.68	c = 168.67
	c = 73.6	c = 73.71	c = 73.00	
Matthew's coef. (Å^3^/Da)	2.02	2.05	2.03	2.22
% solvent	39.2	39.9	39.3	44.7
No. molecules per ASU	2	2	2	4
No. of reflections	190432	192666	168665	148970
No. of unique reflections	27641 (1314)	34457 (1480)	37730 (1469)	28186 (1098)
Multiplicity	6.9 (5.9)	5.6 (3.1)	4.5 (3.2)	5.3 (3.1)
Completeness (%)	98.4 (95.4)	98.4 (85.5)	97.4 (77.8)	91.9 (73.6)
Average mosaicity (°)	0.6	0.4	0.5	0.6
Wilson B factor (Å^2^)	20.3	16.7	14.4	22.3
R_sym_ b (%)	5.7 (40.7)	3.9 (28.0)	3.7 (22.7)	4.2 (22.6)
Mean I/σ(I)	51.5 (5.1)	44.2 (3.0)	40.2 (3.0)	40.6 (3.5)
Refinement				
Resolution (Å)	20.00-1.45	20.00-1.35	20.00-1.30	20.00-1.80
	(1.49–1.45)	(1.39–1.35)	(1.33–1.30)	(1.85–1.80)
R_work_ c (%)	18.5 (27.7)	17.7 (29.8)	17.3 (34.5)	16.2 (25.0)
R_free_ d (%)	22.7 (29.2)	22.3 (30.6)	21.4 (38.4)	23.9 (41.4)
Protein atoms	1358	1407	1435	2739
Ligand atoms	1 (Ca)	1 (Ca)	1 (Ca)	2 (Ca)
No. water	140	215	211	132
Average B factors (Å^2^)	22.8	21.7	18.2	20.9
rmsd Bond length (Å)	0.013	0.014	0.031	0.016
rmsd Bond angles (°)	1.470	1.561	2.036	1.612
**Ramachandran plot**				
Most favoured (%)	95.1	94.0	95.2	93.5
Additionally allowed (%)	4.9	6.0	4.8	6.2
Generously allowed (%)	0	0	0	0.3
Outliers (%)	0	0	0	0

a Values in parentheses are for the highest resolution shell.

b R_sym_ = ∑_hkl_ ∑i [|I_i_ (hkl)−<I(hkl)>|]/∑_hkl_ I_i_(hkl).

c R_work_ = ∑|F_obs_−F_calc_|/∑|F_obs_|, where F_calc_ and F_obs_ are the calculated and observed structure factor amplitudes, respectively.

d R_free_ is the same as R_work_, but 5.0% of the total reflections, chosen at random, were omitted during refinement.

### Structure solution, model building and refinement

The structure was solved by molecular replacement using the structure of a variant of ME ICA512 (Protein Data Bank entry 2QT7.pdb) as a model. Indexing, integration, scaling and reduction were performed with the HKL2000 suite of programs [19]. Five percent of the measured reflections in the high energy dataset were flagged for cross-validation. The initial model was manually completed and refined using COOT [20] and REFMAC5 [21]. The coordinates and structure factors have been deposited in the Protein Data Bank (entries 3N01.pdb, 3N4W.pdb, 3NG8.pdb, and 3NP5.pdb). Molecular graphics were displayed with VMD 1.9 [22] http://www.ks.uiuc.edu/Reserach/vmd/.

### Molecular dynamics

Energy minimizations and simulations were carried out using GROMACS 3.3.3 [23] and GROMOS96 43a1 force field. The initial structures were the energy minimized crystallographic dimers in a solvated rectangular periodic cell. First, a position restrained molecular dynamics run of 40 ps was performed letting water solvent to accommodate to the fixed protein atoms. Second, a 10-ns unrestrained molecular dynamics run was performed. The system was an isobaric-isothermal ensemble at 300 K and 1 bar, with weak temperature coupling (0.1 ps^−1^ respectively). All hydrogen atoms were considered explicitly, and protein covalent bonds were constrained using LINCS [24].

## Results

### X-ray samples and data collection

Two variants of ME ICA512 were prepared for this study. The first comprises residues 470 to 556 and is a major product of the auto proteolysis observed during the crystallization of the full-length ME ICA512 [15]. The second is a point mutant of the first, S508A ME ICA512, and was designed to interfere with the formation of one of the protein-protein interfaces observed previously in the crystal of the wild type protein [15]. The wild type variant was crystallized at pH 8.5 and 4.5; the mutant was crystallized at pH 8.5 and 7.5.

### Unit cells

**Figure 1:**
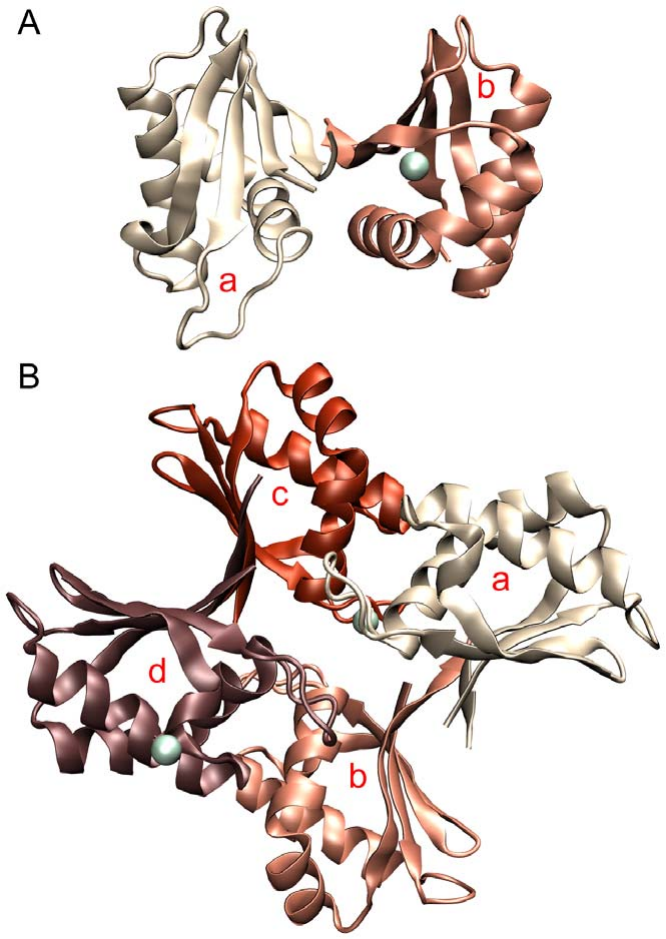
The asymmetric units. Panel A, dimeric assembly found in the orthorhombic unit cells of ME ICA512 crystals grown at pH 7.5 or 8.5. Panel B, tetramer found in the tetragonal unit cells of crystals grown at pH 4.5. Calcium atoms are shown as greenish spheres. Subunits are identified with lower case red letters.

At pH 7.5 or 8.5, ME ICA512 crystallized in the orthorhombic space group *P*2_1_2_1_2_1_ (Table 1). The asymmetric unit comprises two molecules related by a two-fold axis (RMSD from ideal symmetry 0.7±0.1 Å; Fig. 1, Panel A), and the unit cell is essentially the same as in the first described structure of this protein [15].

The molecule at pH 4.5 crystallized in tetragonal *P*4_1_ space group with four chains in the asymmetric unit (Fig. 1, Panel B). In the homotetrameric assembly, subunits AC and BD fairly obey to C2 symmetry (RMSD 1.3±0.1 Å), however all other four monomer combinations significantly deviate from the two-fold symmetry (RMSD 6.1±3.8 Å). As a result, there are no overall symmetry elements other than C1 in the tetramer. Interestingly, dimers AB and CD are two modified versions of the dimer AB found at pH 7.5–8.5 (see below).

### Tertiary structure

The structures solved in this work along with that previously reported [15] allowed the comparison at high-resolution of twelve ME ICA512 protomers found in the different crystals. Despite the differences in the assemblies, mutations, and crystallization conditions, the protomers are remarkably similar with a RMSD of 0.47±0.14 for the backbone atoms. These results confirm that the tertiary structure of ME ICA512 is well preserved under the variety of conditions tested.

ME ICA512 possesses a typical ferredoxin-like (βαββαβ) fold [25], which is characterized by having an antiparallel β sheet covered on one of its faces by two antiparallel αhelices (Fig. 1). This fold is ubiquitous and comprises a large number of superfamilies. Based in a structural and sequence analysis, ME ICA512 pertains to the superfamily of SEA domains [15].

In ME ICA512, the β sheet has regular hydrogen-bond ladders and strand pairing, except for the presence of a β bulge at the beginning of strand β2 and a distortion involving the last and first residues of β1 and β4, respectively. Helix α1 is straight; helix α2 has a characteristic kink that is distinctive of the SEA superfamily. The two helices are amphiphatic, with the hydrophobic faces oriented as to contact each other and the hydrophobic face of the β-sheet.

The hydrophobic core of ME ICA512 is formed by the contacting faces of the helices and the β-sheet. It is a very well packed core with no buried polar residues.

There are two β-turns in ME ICA512. The first pertains to the type II', connects strands β2 and β3, and has glycine and proline at position *i*+1 and *i*+2, respectively. In all the monomers described herein, the proline in this turn adopts a moderately strained phi angle of −86±3.7 degrees. The second turn connects α2 with β4 and is of type I. The remainder regular secondary elements are connected by loops.

### Molecular assemblies

**Figure 2:**
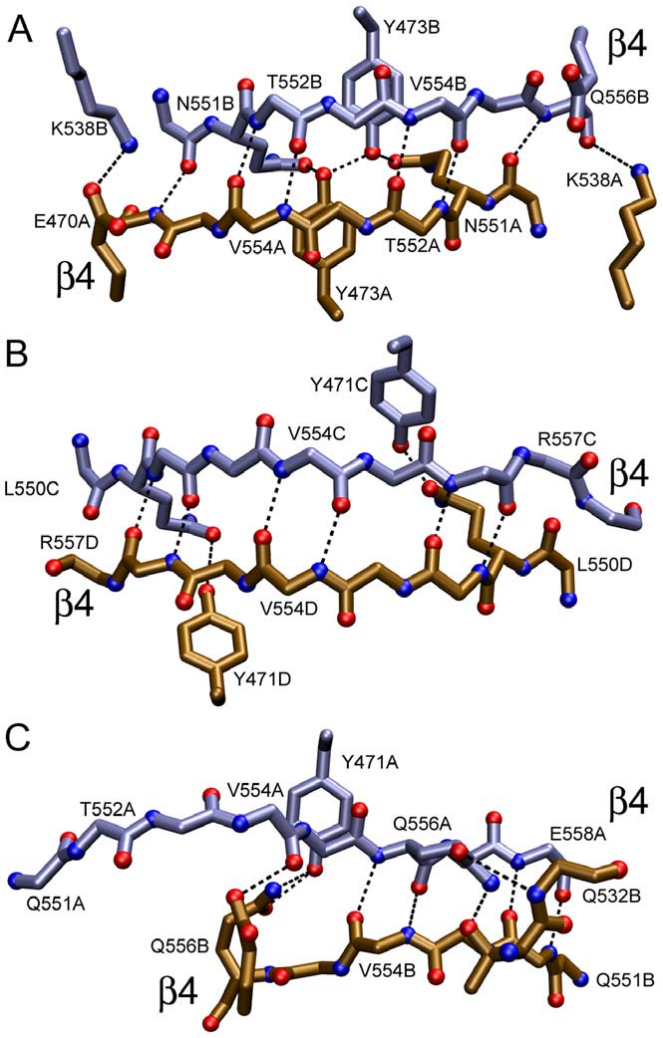
The three β4-β4 dimerization interfaces found in ME ICA512 crystals. Panel A, crystals grown at pH 7.5 or 8.5 and 0.2 mM Ca^2+^. Panels B and C, β4 interfaces formed in the tetrameric asymmetric unit of crystals grown at pH 4.5. To simplify the view, only some of the residues have been numbered. The main-chain, hydrogen-bond bridges are displaced by one and two steps in panels B and C, respectively, compared with Panel A. Side chains involved in hydrogen bonds across the interface are shown.

Examination of the crystal lattices showed several association modes. One of the observed dimer occurs by antiparallel pairing of β4 strands and α2 helices and was named β4—β4 dimer. There are three different versions of the β4—β4 dimer: one in the orthorhombic cell (Fig. 1, Panel A) and the other two in the tetrahedral cell (Fig. 1, Panel B *ab* and *cd*). Two of the β4—β4 interfaces possess C2 symmetry (see panels A and B in (Fig. 2). The remainder is asymmetric (Fig. 2, panel C). A large repertoire of different main- and side-chain hydrogen bonds maintains the strands aligned in different registers with up to 8 Å relative displacements.

**Figure 3:**
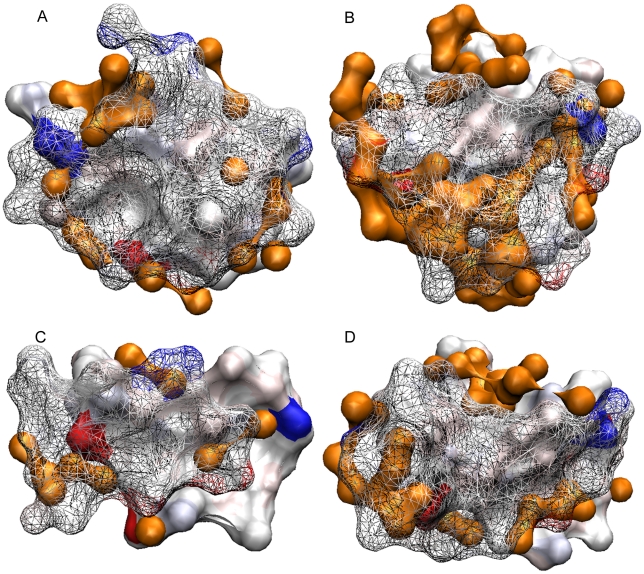
Surface complementarity in the interface of β2—β2 and β4—β4 dimers. Panel A, β2—β2 interface. Panel B, the β4—β4 found in the orthorhombic crystals. Panel C, the β4—β4 interaction between chains A and B in the tetragonal crystal. Panel D, the β4—β4 interaction between chains C and D in the tetragonal crystal. Atoms pertaining at different chains and within 6.5 Å of each other were represented as a surface. One of the chains was rendered solid and the other as a wire mesh to allow the visibility of the underlying surfaces. Strong blue and red colors were assigned to positively and negatively charged atoms, respectively. Uncharged atoms are depicted accordingly to polarity (pink oxygen, light blue nitrogen, and grey carbon). The surface of water molecules is shown in orange. The page plane bisects the dimers, and the interacting β-strands run horizontally.

The β4—β4 interface in the orthorhombic assembly buries ∼1100 Å^2^ of monomer surface. The other two, in the tetragonal assembly, involve 800–900 Å^2^ each. A visual inspection shows good surface complementarity and with the exception of the orthorhombic β4—β4 interaction, also tight solvent exclusion (Fig. 3).

**Figure 4:**
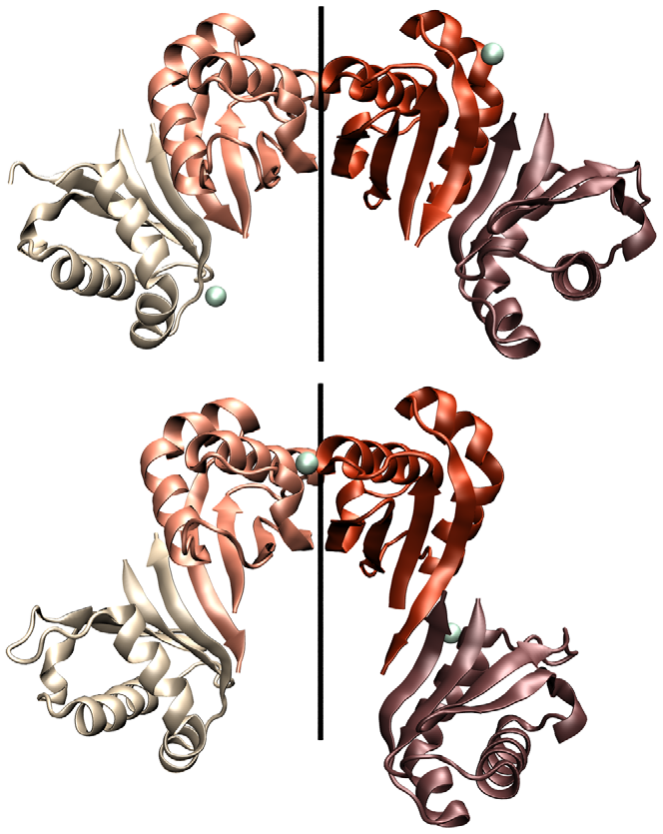
The alternating β4—β4/α2—α2 tetramers. Panel A, structures at pH 7.5–8.5. Panel B, structure at pH 4.5. Calcium atoms are shown as greenish spheres. The two-fold axis for the two central equivalent β2—β2 dimers are shown.

A second dimerization mode occurs through the antiparalell pairing of β2 strands. In the orthorhombic cells β2—β2 and β4—β4 pairing alternate forming a continuous, antiparallel β sheet with a two-fold screw axis along the *c* cell dimension (Fig. 4, Panel A). A similar β2—β2 and β4—β4 pattern arises in the tetrahedral cells by translation of the tetrameric asymmetric unit along the axes *a* or *b* (Fig. 4, Panel B). However, as mentioned above, there are two different β4—β4 interfaces in the tetrahedral cell.

**Figure 5:**
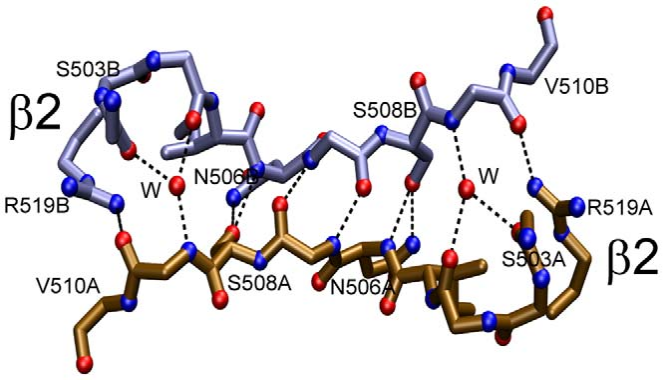
The β2—β2 dimerization interface. This interface is nearly identical in the crystals grown at pH 4.5 (3NP5.pdb) or at pH 7.5 and 8.5 (2QT7.pdb, 3NOI.pdb, 3NG8.pdb, 3N4W). Two symmetrically disposed water molecules (W) contribute maintaining the hydrogen bonding between the divergent strands.

The β2—β2 interface buries ∼1200 Å^2^ and involve ten hydrogen bonds and numerous van der Waals contacts (Fig. 5). Surface complementarity inspection indicates good fitting and water exclusion at the interface (Fig. 3, Panel A).

Due to a β2 bulge formed by Asn 506, the two β2 strands in the edge have a strong convex bend and therefore cannot efficiently align backbone O and N atoms to form regular hydrogen bonds across the dimer interface. As a consequence, there are only two hydrogen bridges between backbone atoms. The lack of main-chain register is compensated by using subrogate side chain N and O atoms to mimic the standard antiparallel bridging: Asn 506, Ser 508, and Arg 519 (the latter from strand β3) all bridge across the interface and form elaborate and multiple hydrogen bonds among them and with main chain atoms. Due to the two-fold symmetry, each interaction is duplicated. All of the potential hydrogen bonds in the main chain are so realized, except those between Phe 504 and Val 509. Remarkably, these hydrogen bond, which cannot be formed directly due to the divergence of the strands, are realized through two water molecules conserved in all the five solved structures of ME ICA512.

**Figure 6:**
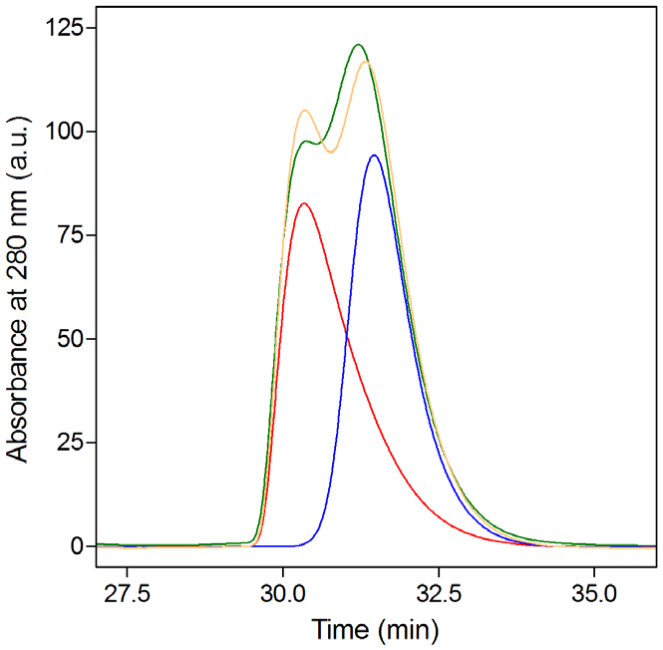
Size exclusion chromatography. Analytical size exclusion chromatography of ME ICA512. At the concentration tested, wild-type ME ICA512 behaves as a dimer (red trace, [15]). S508A ME ICA512 is monomeric (blue trace). Co-chromatography of the two proteins (green trace) produces two overlapping peaks that are similar to the algebraic sum (yellow trace) of the red and blue traces. Each variant was injected at the same concentration (500 µM) in 100 µl of elution buffer (see Methods). This result indicates that heterodimers are not significantly populated at equilibrium in the mixture. Since the X-ray structures of the two variants are nearly identical, Ser 508 must be directly involved in the associating surfaces.

Most important, site specific mutagenesis combined with size exclusion chromatography and light scattering measurements confirmed that the β2—β2 dimer is populated in solution (not shown). Indeed, the variant S508A was originally designed based on the first published structure of ME ICA512 (PDB entry 2qt7) aiming to specifically weaken the binding at the β2—β2 interface. Unlike the wild type protein that associates in a concentration dependent manner [18], S508A is monomeric and well-folded at all concentration tested. The same happens with another mutant designed to have a weakened β2—β2 interface, I507P. On the contrary, a mutation designed to disturb the β4—β4 interface, G553D, has no effect on the dimeric behavior of the protein (Fig. S1 in Supporting Information). The impact of the mutation S508A on the hydrodynamic behavior of ME ICA512 is further illustrated in Fig. 6. When injected into the calibrated size exclusion chromatography column separately, wild-type and mutant proteins originate well resolved peaks corresponding to dimer and monomer, respectively. When preincubated and injected together, the two proteins originate a trace that match the sum of the peaks obtained running the proteins separately. This result indicates that at equilibrium, the concentration of heterodimer formed by association of wild-type and mutant subunits is very low, confirming that the main association mode in solution involves the β2—β2 interface.

**Figure 7:**
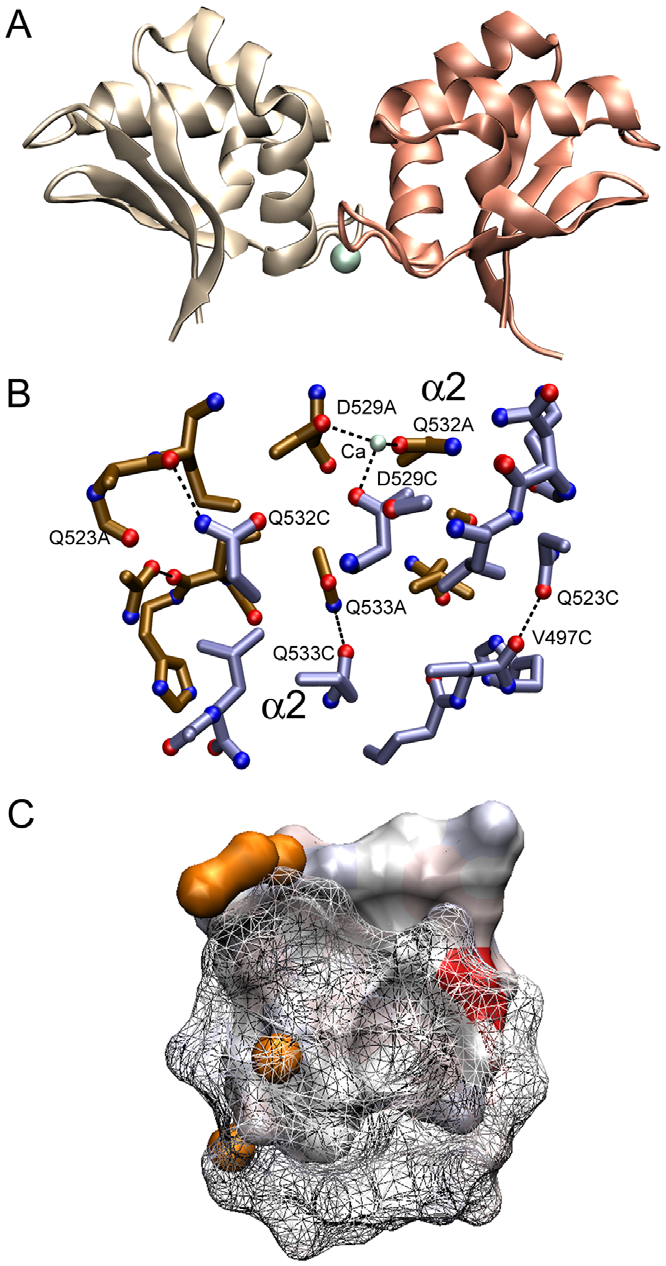
Interaction α2—α2 found in the tetrameric asymmetric unit of crystals grown at pH 4.5. Panel A. Overall view. Panel B. Main contacts between the two interacting monomers. Only some of the residues are shown to simplify the view. Calcium is represented as a greenish sphere. Panel C. The surfaces occluded by the dimer represented as described in the legend to Figure 3.

A third kind of dimer, present exclusively in the tetrahedral cell, is formed by the interaction of α2 helices (Fig. 7). There are two dimers of this kind, AC and BD in the tetrahedral asymmetric unit (see Fig. 1, Panel B). This dimer buries ∼800 Å^2^ of molecular surface. Unlike the interactions between β-strands, the α2—α2 interface is very rich in hydrophobic contacts. It includes however four hydrogen bonds and in one of the two dimers it also includes a Ca^2+^ binding site (see below) across monomers.

Although there are several other contacts between monomers in the lattices of the different crystals of ME ICA512, those described above are the only with two fold symmetry and with a sufficiently large buried surface as to be considered possible biologically relevant interfaces. In this regard, the evolutionary conservation of residues in these interfaces was examined by performing an alignment of 22 sequences of ICA512 and phogrin from different organisms. The result indicates that nearly invariant residues engage in the formation of the β4—β4 interface, whereas β2—β2 and α2—α2 are formed by much more variable sequences (see Fig. S2 and S3 in the Supporting Information).

To obtain further insight into the relevance of the interfaces described herein, 10-ns molecular dynamic simulations were performed (not shown). The molecules included in the simulation were the α2—α2, β2—β2, β4—β4 dimers. Two additional monomer-monomer interactions with small buried surface and present in the crystal lattice were tested as a control. In all cases, the tertiary structure of the monomers was well-preserved throughout the runs. The crystal interactions were rapidly disrupted in the control runs in the case of α2—α2. On the contrary, dimers β2—β2 and β4—β4 were stable throughout the simulations. However, whereas the β2—β2 strand register and hydrogen bonding described in Fig. 5 varied little and was present most of the time, the interaction for β4—β4 was much more fluctuating. Interestingly, the behavior of β4—β4 interface in the simulation is reminiscent of the plasticity demonstrated in the crystal (see Fig. 2).

### Metal binding sites

CaCl_2_ is present at high concentrations in the crystallization milieu (0.2 M), and several calcium atoms were included in the refined structures. Since the purified protein contains no significant amounts of metals [18] and in each occurrence Ca^2+^ coordinates three or less protein atoms from different chains, binding seems the result of crystal packing rather than of pre constituted intra-chain binding pockets. One of the Ca^2+^ binding sites is present in all the crystals obtained at pH 7.5 or 8.5 and involves a bidentated carboxylate, a main-chain oxygen from one chain, and a ND1 atom from an histidine residue in another chain (Fig. S4, Panel A in Supporting Information). Another Ca^2+^ binding site is exclusive of the pH 4.5 structure and it contributes to the interface established between chains A–C in the tetrameric asymmetric unit (Fig. S4, Panel B in Supporting Information). The first resolved structure of ICA512 (at pH 8.5; [15]) contained calcium bound to a site that appears as empty in the new structures reported herein. This calcium atom is also coordinated by three protein ligands from two different chains (not shown). Finally, and for completeness, in the crystals obtained at pH 4.5 a calcium atom is found at coordinating distance of the OG of serine 500. However, no other protein atom is coordinated to this calcium and its significance is unclear.

From the analysis of bound calcium a short list of metal-binding residues of ME ICA12 was elaborated. Interestingly, all of these residues cluster into two close protein surface region: the loop connecting α2 and β4, and the beginning of α2. This finding might guide the search of potential interfaces for metal-mediated ME ICA512 interactions in a biological context.

## Discussion

ICA512 is a very complex molecule with several domains and conceivably multiple functions. It is clear that the molecule is extensively processed and that during its life cycle its parts become exposed to very different cellular and tissue contexts, such as the nucleus, cytoplasm, lumen of organelles along the secretory pathway, membrane compartments, and the extracellular pancreatic islet space. Here, we focus on ME ICA512, a portion of the receptor that is first within the endoplasmic reticulum and the Golgi cisternaes, subsequently in the SG lumen where it is exposed to further changes in the local H^+^, Ca^2+^, Zn^2+^, and Cl^−^ concentrations, and finally faces the extracellular space and returns to a neutral pH [7].

The X-ray structures of ME ICA512 solved for this work and the one we reported previously [18] sample the extreme pH conditions the fragment encounter in its journey from the trans-Golgi network to the extracellular space. The fact that the tertiary structure of ME ICA512 is nearly identical in all those cases establishes that no significant conformational changes are associated with the pH changes. Of particular interest is also the finding that Ca^2+^ binding to ME ICA512 is likely due to inter-chain contacts induced by crystallization and not to specific intra-chain binding sites of the kind that are usually functional. Nevertheless, in view of what is observed for other prominent SG proteins [7], the possibility that the residues that coordinate Ca^2+^ in the ME ICA512 crystals contribute to metal-binding induced aggregation or multimerization of ME ICA512 in vivo cannot be entirely dismissed.

The quaternary structure of ME ICA512 is also of considerable interest. There is evidence that the intracellular and the transmembrane regions of ICA512 have oligomerization potential per se, and it has been proposed that association processes mediate several of the functions of this protein [7]. Indeed, homo and hetero dimerization are at the heart of many biological functions mediated by type I membrane receptors and these phenomena are considered particularly important in the RPTP family of proteins.

Most, if not all, atomic models of receptors oligomers are based on X-ray evidences obtained by crystallizing isolated soluble domains and then deducing the structure of the whole from the structure of the parts. This is so because of the almost insurmountable experimental difficulties involved in the determination of the X-ray or NMR structure of a membrane embedded oligomerized receptor. Specifically, crystallographic interactions between isolated domains, frequently mimics association modes found or assumed to exist in complete receptors (see for instance references [26], [27]).

The crystal structures of ME ICA512 reveal five potential surfaces for receptor dimerization. All have a two-fold axis of symmetry, as almost all biologically relevant dimers do. One of these, the β2—β2 dimer (Fig. 4 and 5), occurs in solution, since mutations designed to perturb the equivalent interface (S508A, I507P) shift the equilibrium toward the monomer. Interestingly, the perturbation of the equilibrium in the case of S508A ME ICA512 does not impede the formation of the β2—β2 interface in the crystal and the structures of wild type and S508A ME ICA512 are nearly identical. Thus, the β2—β2 interaction is a strong candidate for biological relevance.

A second kind of dimers revealed in the crystals is that mediated by the β4—β4 interactions. Three different but related forms of this interaction were observed. These forms exhibit different hydrogen bond register between β-strands. Two of them have a two-fold symmetry (Fig. 2 and 4). We have shown that the major dimer population in solution involves β2—β2 pairing. Therefore the β4—β4 dimer, if ever formed, must have low affinity. Direct inspection and standard computational analysis of the interfaces of the β4—β4 dimers in the crystals also suggest a weaker interaction compared with β2—β2 association. Judging from the molecular dynamics results, the hydrogen bonding across the interface is less rigid and more fluctuating than in the β2—β2 interface. This, along with the existence in the crystal lattice of three different variants of the β4—β4 interaction, suggests that the latter might be relevant in vivo for the formation of complementary or more promiscuous associations. Nevertheless, the differences in stability between the two complexes are unlikely to be large, because both are preserved in the molecular dynamic simulations.

The fifth potential dimerization interface revealed by the crystal structures is that established at pH 4.5 through the first half of the α2 helix. Based on the standard computational analysis and using the same considerations applied in the previous case, the association constant for this dimer should be small; also, since this interaction is not preserved in the molecular dynamics runs, it appears to be less stable than the β4—β4 association mode.

Even though the β4—β4 and α2—α2 dimers seem to have low potential for dimerization in solution, they can not be discarded as leads for the associations that the entire receptor might undergo in vivo. Admittedly, crystallization can stabilize interactions modes that are not biologically relevant; however, there is a strong tendency for C2 symmetry in low-energy dimers [28], and it is unlikely that crystallization would have stabilized high-energy interactions. Thus, the crystallization of ME ICA512 likely recapitulates favorable association modes for this molecule. Otherwise, association would be competing with crystallization. If additional factors were present, as for instance a topological restrain imposed by the geometry of the whole receptor in the membrane, any of these dimers would be further stabilized and become significantly populated.

A further observation must be taken into account in the discussion of the biological relevance of the interactions modes observed in the crystals of ME ICA512. Alignment of ICA512 and phogrin sequences using the solved structures as a guide shows a very strong sequence conservation of the residues interacting in the β4—β4 complexes, whereas all the other interaction modes described herein involve residues much less conserved. This behavior is consistent with a biological role for the surface occluded in the β4—β4 complexes, and since protein-protein interacting surfaces in biological complexes tolerate far less evolutionary changes than the solvent exposed surfaces, this biological role might be mediating the receptor dimerization.

In summary, we have described the structure of ME ICA512 under a variety of solution conditions relevant for the biological function. Also a catalog of interacting surfaces candidates for biological relevance and their properties is provided. These pieces of information are necessary for the modeling of the whole receptor embedded in a membrane and for the design of experiments aimed to prove the structural and functional hypothesis that such model will suggests.

## Supporting Information

Figure S1 R_s_ of ME ICA512 mutants as a function of protein concentration. Circles represent the experimental results for the wild type protein (black), S508A (red), I507P) (green) G553D (blue). The line represents the least-square fit of a monomer-dimer equilibrium equation to the data of the wild type protein (K_D_ = 0.8 µM). I507P and S508A are mutants that perturb the β2—β2 interface. G553D alters the β4—β4 interface. The concentration axis indicates the final average concentrations in the eluting peak at equilibrium (i.e., samples were injected in the column at much higher concentrations and undergo a diffusion-mediated dilution during chromatography.
(TIF)

Figure S2 Alignment of ICA512 (*) and phogrin (**) sequences of mature ectodomains. Residues identical in all sequences are in dark green columns. Light green columns indicate identity in more than 90% of the positions.
(TIF)

Figure S3 Residues identical in >90% of the sequences aligned in Fig. S1 are shown in green. For the different association modes observed in the crystal lattices, one of the interacting subunits was represented as a surface and the other as a cartoon. Panel A, β4—β4 dimer. Panel B, β2—β2 dimer. Panel C, α2—α2 dimer.
(TIF)

Figure S4 CPK representation of Ca^2+^ binding sites. Panel A. Binding to monomers A and D in the tetragonal crystals. Panel B. Binding to chains A and B in the orthorhombic crystals. Calcium atoms are shown as greenish spheres.
(TIF)
