# Supplementary figures and images for: Protein-Protein Interactions in Crystals of the Human Receptor-Type Protein Tyrosine Phosphatase ICA512 Ectodomain
Source: PLoS One. 2011 Sep 15;6(9):e24191. doi: 10.1371/journal.pone.0024191 (PMC3174154; doi:10.1371/journal.pone.0024191)

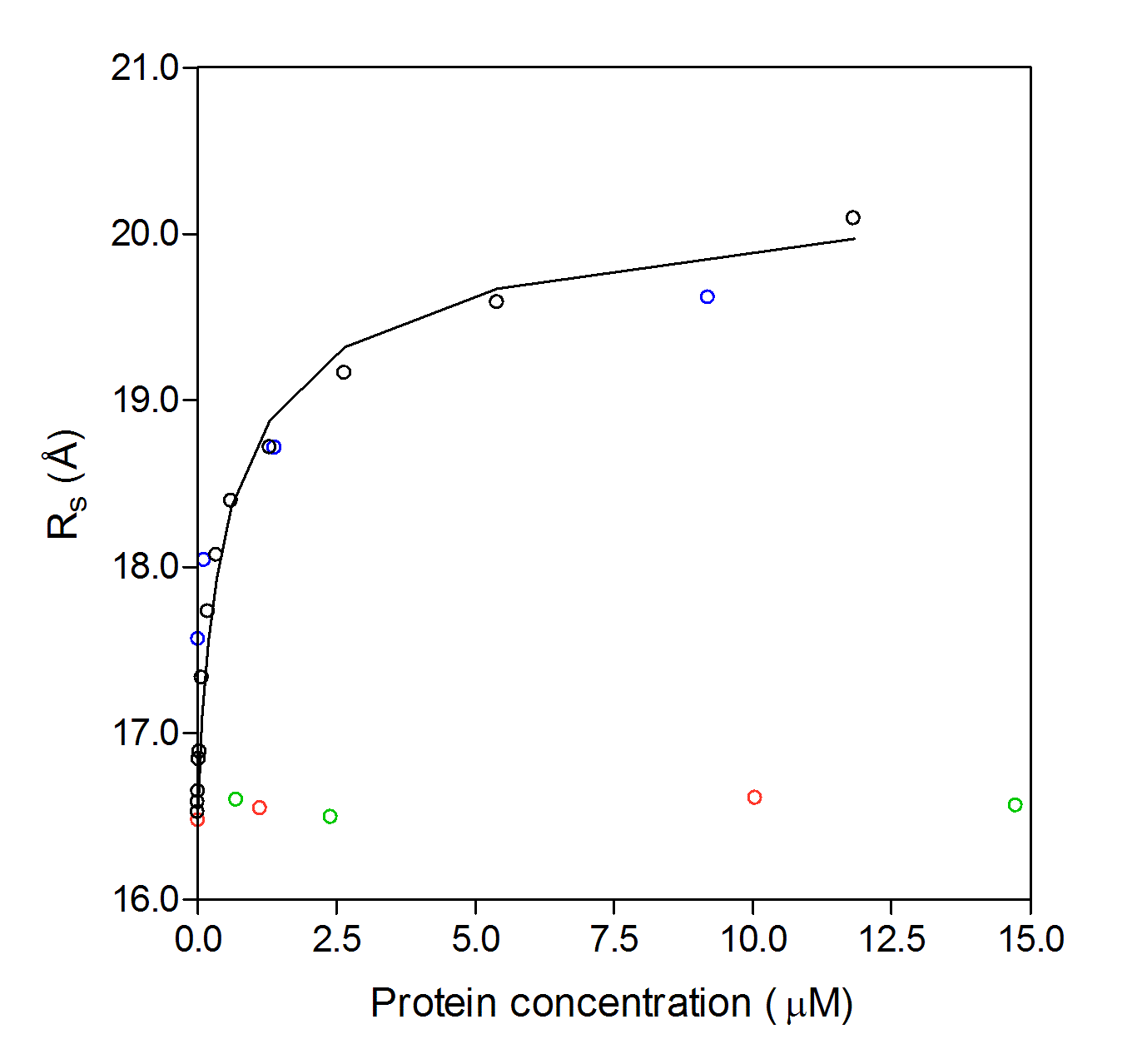

Supplement: Figure S1 — Rs of ME ICA512 mutants as a function of protein concentration. Circles represent the experimental results for the wild type protein (black), S508A (red), I507P) (green) G553D (blue). The line represents the least-square fit of a monomer-dimer equilibrium equation to the data of the wild type protein (KD = 0.8 µM). I507P and S508A are mutants that perturb the β2—β2 interface. G553D alters the β4—β4 interface. The concentration axis indicates the final average concentrations in the eluting peak at equilibrium (i.e., samples were injected in the column at much higher concentrations and undergo a diffusion-mediated dilution during chromatography. (TIF) [file pone.0024191.s001.tif]

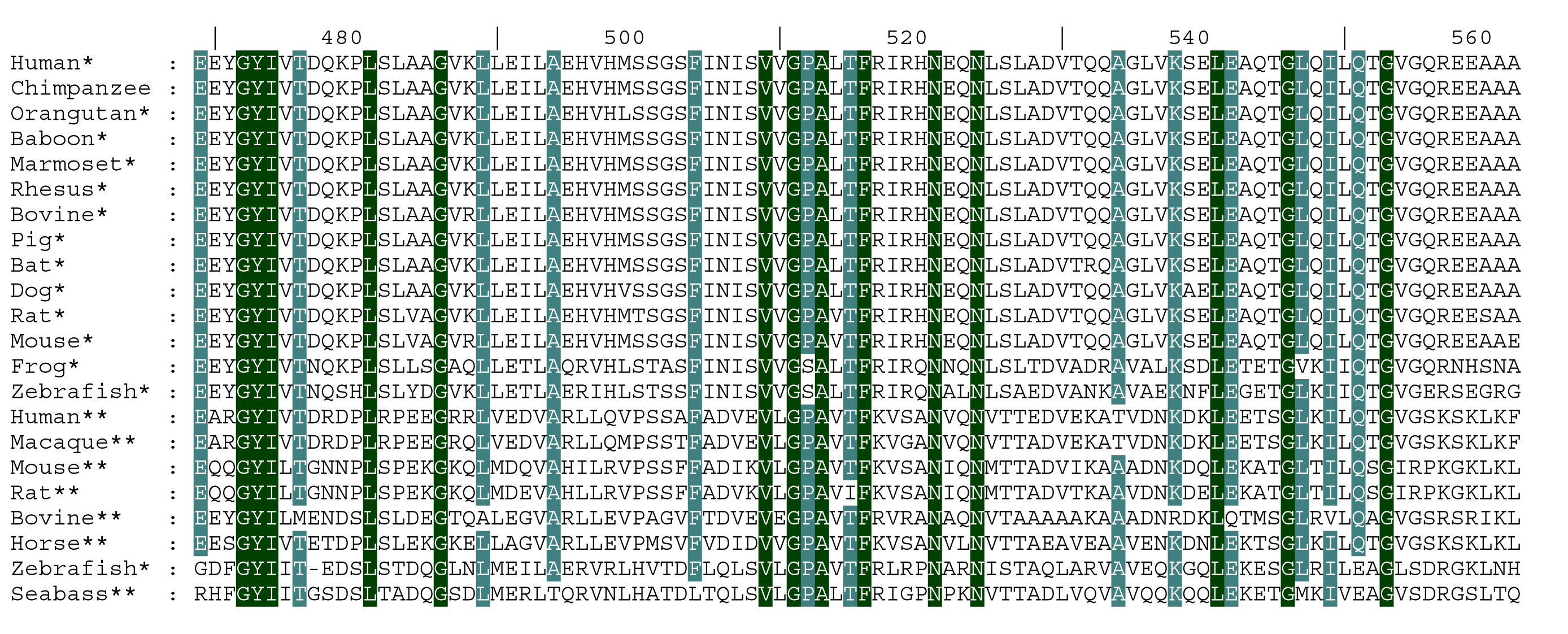

Supplement: Figure S2 — Alignment of ICA512 (*) and phogrin (**) sequences of mature ectodomains. Residues identical in all sequences are in dark green columns. Light green columns indicate identity in more than 90% of the positions. (TIF) [file pone.0024191.s002.tif]

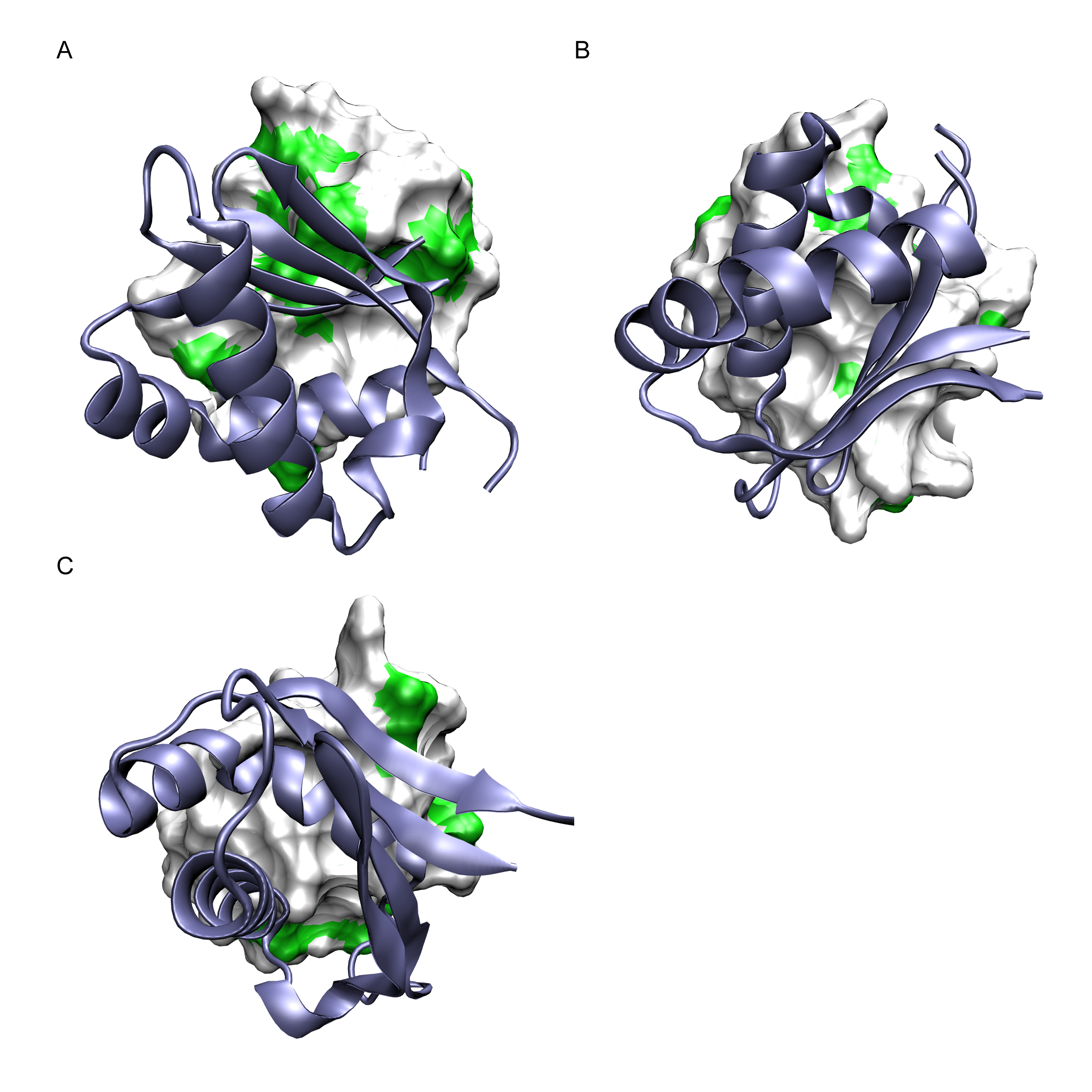

Supplement: Figure S3 — Residues identical in >90% of the sequences aligned in Fig. S1 are shown in green. For the different association modes observed in the crystal lattices, one of the interacting subunits was represented as a surface and the other as a cartoon. Panel A, β4—β4 dimer. Panel B, β2—β2 dimer. Panel C, α2—α2 dimer. (TIF) [file pone.0024191.s003.tif]

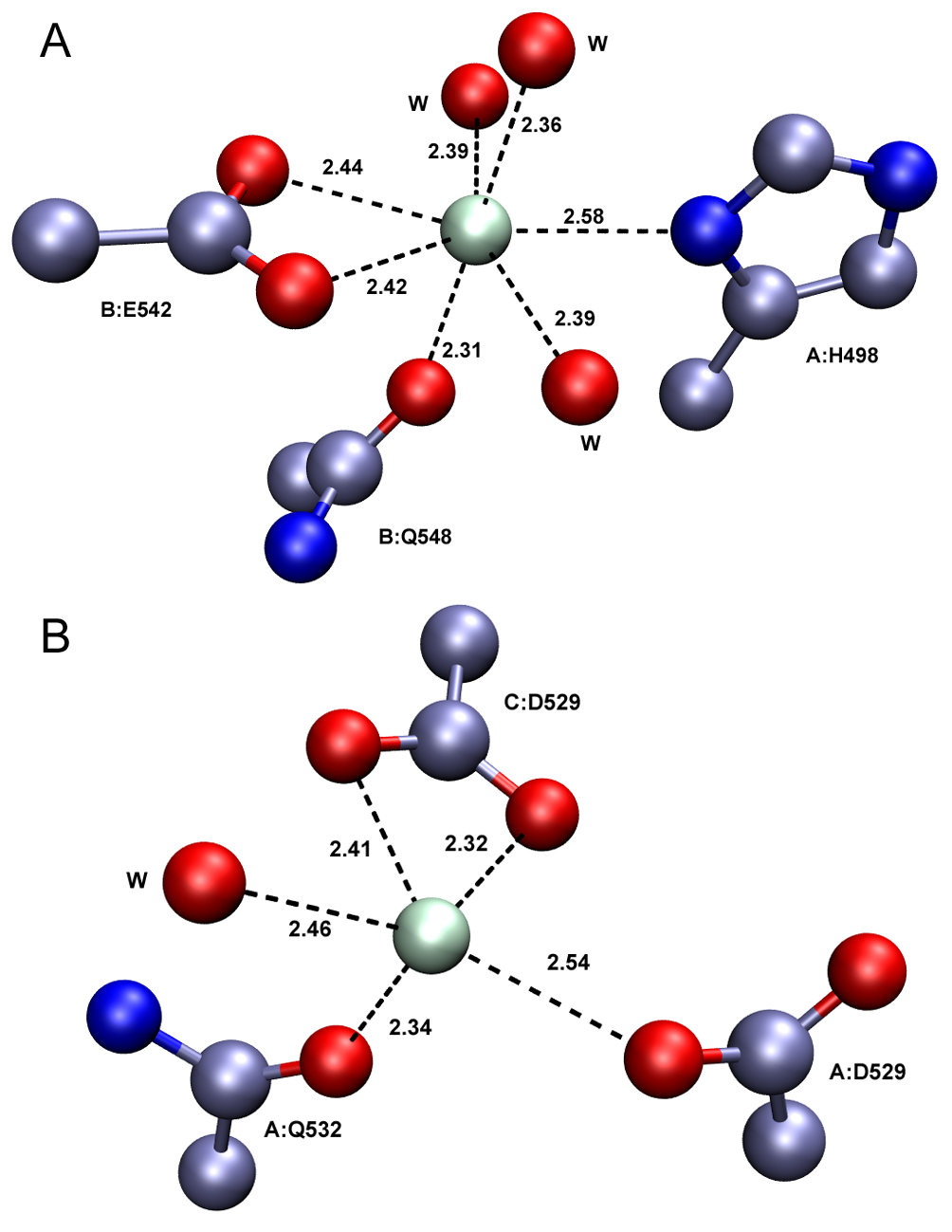

Supplement: Figure S4 — CPK representation of Ca2+ binding sites. Panel A. Binding to monomers A and D in the tetragonal crystals. Panel B. Binding to chains A and B in the orthorhombic crystals. Calcium atoms are shown as greenish spheres. (TIF) [file pone.0024191.s004.tif]
